# Reducing the motor response in haptic parallel matching eliminates the typically observed gender difference

**DOI:** 10.1007/s00221-015-4437-z

**Published:** 2015-09-16

**Authors:** Hanneke I. van Mier

**Affiliations:** Department of Cognitive Neuroscience, Faculty of Psychology and Neuroscience, Maastricht University, P.O. Box 616, 6200 MD Maastricht, The Netherlands

**Keywords:** Haptic perception, Egocentric, Allocentric, Gender, Frame of reference, Parallelity

## Abstract

When making two bars haptically parallel to each other, large deviations have been observed, most likely caused by the bias of a hand-centered egocentric reference frame. A consistent finding is that women show significantly larger deviations than men when performing this task. It has been suggested that this difference might be due to the fact that women are more egocentrically oriented than men or are less efficient in overcoming the egocentric bias of the hand. If this is indeed the case, reducing the bias of the egocentric reference frame should eliminate the above-mentioned gender difference. This was investigated in the current study. Sixty participants (30 men, 30 women) were instructed to haptically match (task HP) the orientation of a test bar with the dominant hand to the orientation of a reference bar that was perceived with the non-dominant hand. In a haptic visual task (task HV), in which only the reference bar and exploring hand were out of view, no motor response was required, but participants had to “match” the perceived orientation by verbally naming the parallel orientation that was read out on a test protractor. Both females and males performed better in the HV task than in the HP task. Significant gender effects were only found in the haptic parallelity task (HP), corroborating the idea that women perform at the same level as men when the egocentric bias of the hand is reduced.

## Introduction

A seemingly simple task as making two bars haptically parallel to each other has been found to result in (often) large deviations. In such a task, blindfolded participants have to match the orientation of a reference bar that is felt with one hand, on a test bar that is rotated with the other hand. What feels as being parallel in this task is often far from what is physically parallel, suggesting that the haptic perception of spatial relations is not veridical (Kappers 1999; Henriques et al. 2004). Based on the results in reaching and grasping tasks, Flanders and Soechting (1995) suggested that hand orientation is controlled in an “intermediate” frame of reference. Following this suggestion, Kappers (2002, 2003) proposed that the deviations observed in haptic parallelity are most likely also caused by the use of a frame of reference that is intermediate to a frame based on egocentric and allocentric information. An egocentric reference frame refers to a frame that is centered on the body, while an allocentric reference frame is linked to external space (Klatzky 1998). While the use of an allocentric reference frame would result in bars that would be perfectly parallel, the use of an egocentric reference frame would result in large differences between the orientations of both bars. The observed deviations are usually intermediate between allocentrically and egocentrically parallel and as such support a reference based model for haptic parallel matching (Van Mier 2014).

Deviations have been found to increase when the horizontal distance between the hands is increased (Fernández-Díaz and Travieso 2011; Kaas and Van Mier 2006; Kappers 1999; Van Mier 2013; Zuidhoek et al. 2003), but not when the distance is changed vertically (Fernández-Díaz and Travieso 2011; Kappers and Koenderink 1999). For most participants, deviations are larger when oblique orientations have to be paralleled compared to cardinal orientations (Kaas and Van Mier 2006; Kappers 1999, 2002, 2003; Kappers and Viergever 2006; Van Mier 2013; Zuidhoek et al. 2005), the so-called haptic oblique effect. However, for participants with large deviations, a reversed oblique effect has been observed (Hermens et al. 2006; Kappers 2003, 2004; Volcic et al. 2007). These participants showed larger deviations for the cardinal than for the oblique orientations. For these participants, who rely more on an egocentric frame of reference, the physically oblique orientations are more or less aligned with the orientation of the hand and are therefore non-oblique with respect to the egocentric reference frame, while the physically cardinal orientations are considered oblique. When the hands are positioned farther away from each other in the horizontal direction, the orientation of the hands is different. This suggests that the deviations in haptic parallelity matching are most likely the result of an egocentric reference frame that is biased by the hand (Kappers 2002, 2003; Van Mier 2014). Several authors have reported results that support a hand-centered egocentric reference frame in haptic parallelity tasks (Kappers and Liefers 2012; Kappers and Viergever 2006; Van Mier 2013; Volcic and Kappers 2008). Furthermore, the distortions in parallelity are not random, but show a systematic bias in the direction of the natural orientation of the hand, being mainly in clockwise direction when the test bar is on the right side and in counterclockwise direction when the test bar is on the left side (Kappers 2004).

Results of several studies support the idea of an intermediate reference frame in haptic parallel matching. It has been found that deviations were significantly smaller when a delay was introduced between the perception of the orientation of a reference bar and the parallel matching of this orientation on a test bar (Postma et al. 2008; Zuidhoek et al. 2003, 2004a, 2007). It was proposed that the introduction of a delay resulted in a shift from an egocentric toward a more allocentric reference frame during the delay. It was hypothesized that during the delay a visual image of the perceived orientation was formed, increasing the contribution of the allocentric reference frame. Evidence for this hypothesis was found in an fMRI study performed by Kaas et al. (2007a), showing activation in parieto-occipital cortex later in the delay period, an area known to be involved in visual imagery. Another manipulation that has been shown to improve haptic parallel setting is providing (non)-informative vision. In these studies, participants could freely look around while the bars and their hands were out of view (non-informative vision: Newport et al. 2002; Volcic et al. 2008; Zuidhoek et al. 2004b) or could only see the test bar and their matching hand (informative vision: Van Mier 2013). It was hypothesized that vision stimulated the use of an allocentric reference frame. In line with the intermediate reference frame model, reduction in the bias of the hand-centered egocentric reference frame resulted in improved haptic parallelity performance (Van Mier 2013). These results show that when the weight of the reference frame is changed, performance changes as well.

Research has shown that the size of the deviations is participant-dependent, with a large variation between participants (e.g., Kappers 2003, 2004; Van Mier 2014; Volcic et al. 2008; Zuidhoek et al. 2003). Regardless of this large inter-individual variation, a persistent finding has been that males outperform females on haptic parallel matching tasks (Hermens et al. 2006; Kaas and Van Mier 2006; Kappers 2003, 2007; Van Mier 2013; Volcic et al. 2008; Zuidhoek et al. 2007). Kappers (2003) showed that the advantage of males in haptic parallel performance was not due to their educational or professional experience. Even when controlling for these factors, men had smaller deviations than women (Kappers 2003). The gender difference in performance may be accounted for by different contributions of the ego- and allocentric reference frames in women and men. Based on the fact that women showed larger egocentric weighting factors than men, Kappers (2007) suggested that women might be more egocentrically oriented than men. However, Zuidhoek et al. (2007) found that a shift from the use of an ego- to a more allocentric reference frame as a result of including a 10-s delay between perception and matching resulted in improved performance that was similar in men and women. According to the authors, this finding suggests that women not necessarily rely less on an allocentric reference frame than men, but that they are less efficient in overcoming the egocentric bias of the hand when haptically matching the orientation of a bar. Evidence for this line of thought that stimulating the allocentric processing of haptic parallelity matching benefits males and females to the same extent comes from a study by Van Mier (2013). In this study in one of the conditions, participants had full view of the test bar and their matching hand, while the view of the reference bar and their exploring hand was blocked. In this condition, participants could use external visual cues of the setup and the environment, like the sides of the plate with the protractor and bar or the table, or the walls and doors. These cues most likely stimulated the use of allocentric processing, as suggested by a highly significant reduction in deviations. Although males still performed significantly better than females in this condition compared to haptic parallel matching, the reduction was similar in both genders. Performance of males and females did not significantly differ when they were instructed to match the orientation of a bar to a verbally given clock time (Zuidhoek et al. 2007). However, when the same participants had to feel the orientation of a bar and had to report this orientation as a clock time, women had significantly larger deviations than men. Although the instructions in both conditions stimulated the use of an allocentric reference frame, in the latter condition performance was more biased by a hand-centered reference frame than in the former (see also Van Mier 2014). Because women are thought to be less able to reduce the egocentric bias of the hands, their performance deviated more from veridicality in the above-mentioned studies and conditions than the performance of the male participants.

In the haptic parallel studies in which gender-related differences in deviations were reported (Hermens et al. 2006; Kaas and Van Mier 2006; Kappers 2003, 2007; Van Mier 2013; Volcic et al. 2008; Zuidhoek et al. 2007), participants had to actively match the orientations using their hands. Additionally, there was a horizontal distance between the bars and hands in these studies. It has been established that increasing the distance between the bars/hands results in larger deviations (Fernández-Díaz and Travieso 2011; Kaas and Van Mier 2006; Kappers 1999, 2002, 2003; Kappers and Koenderink 1999; Van Mier 2013; Zuidhoek et al. 2003). As Kappers and Viergever (2006) stated, when the two bars are horizontally apart, the orientations of the two hands and arms will be quite different and the deviations will be dependent on the orientation of the hand. Egocentric participants do not sufficiently take into account the orientation of their hands. Participants with larger deviations (in particular women) will be more influenced by the difference in orientation of the hands due to the distance between the hands. This suggests that in conditions in which the horizontal difference between the bars and hands is zero or in which the egocentric bias of the hand is highly reduced or even absent, performance of female and male participants should not be significantly different. This has indeed been reported. In three studies in which the interfering bias of the egocentric reference frame was considerably reduced because there was no distance between the hands (Kappers and Liefers 2012), because the orientation had to be drawn (Van Mier 2013), or because matching was done in a passive mode (Kappers and Schakel 2011), males and females performed at the same level. In the haptic parallelity study by Kappers and Liefers (2012), the test and reference bar were located directly in front of the participant at the same location horizontally and only differed in the vertical direction (one hand performed above the other). In this task, the hands performed at the same location with a distance of zero in the horizontal plane. Systematic deviations were found as a function of the angle between the hands showing an egocentric bias involving the hand. However, there was no (additional) difference in orientation between both hands due to a distance between the bars and hands, and therefore, this interfering effect was not present in this study. Results showed that the performance of females and males was not significantly different in this task. Minimizing additional interference of hand orientation by eliminating the distance between the hands might have accounted for the nonsignificant gender difference in the study by Kappers and Liefers (2012). Additionally, when the distance between the stimuli increases, the distance to the body increases as well, suggesting an influence of a body-centered frame of reference. Studies have shown that both hand- and body-centered egocentric reference frames play a role in haptic parallel matching, although the hand-centered reference frame has been found to be the most influential (Kappers 2007; Kappers and Viergever 2006; Volcic et al. 2009). It is possible that women are more influenced by the egocentric body-centered reference frame than men. Alternatively, performance of females and males did not significantly differ in the study of Kappers and Liefers (2012) because the influence of the body-centered reference frame was zero since the stimuli were presented at the same location with respect to the body. However, caution is needed interpreting the nonsignificant gender differences in this study because only 12 participants (6 of each gender) were included in the study. In the study by Van Mier (2013), the egocentric bias of the hand was decreased by having participants draw the matched orientation instead of rotating the bar. In this condition, participants had only full view of their drawing hand, while the hand that perceived the orientation of the reference bar was blocked from view. In this condition, the drawing movements were performed by the fingers of the hand holding a pencil, but were directed from the arm/shoulder and thereby reduced the bias of the hand. This resulted in deviations for males and females that were not significantly different. Kappers and Schakel (2011) included a condition in their study in which the use of the hands was completely eliminated. In this condition, participants had full view of the setup and both bars and did not orient the test bar themselves but instructed the experimenter to set the test bar in such a way that it paralleled the orientation of the reference bar. Also in this condition, no significant difference in performance was found between male and female participants.

The results regarding nonsignificant gender differences so far have been based on parallelity tasks in which no hand movement was required (Kappers and Schakel 2011) or where the bias of the matching hand was reduced (Van Mier 2013). Based on these findings, no gender differences would be expected in a parallelity task in which only hand movements were required when perceiving the orientation but not when matching the orientation. This was explored in the current study in which male and female participants performed such a task in addition to a haptic parallelity task. As in the study by Van Mier (2013), the reference bar and the perceiving hand were out of view. The plate with the protractor and test bar was replaced by a plate with a protractor containing letters and numbers for each orientation from 0° to 270°. Participants had to match the orientation of the reference bar on the test protractor by verbally stating which orientation was parallel to the felt orientation by naming the corresponding letter–number combination. Because no hand movement was required when verbally “matching” the orientation, we anticipated no gender differences in this condition, contrary to the haptic parallel task, where significant gender differences were expected.

## Methods

### Participants

A total of 60 participants were tested in this study, 30 females (mean age 35.4 years, SD = 14.4, age range 18–62 years) and 30 males (mean age 37.3 years, SD = 14.9, age range 19–62 years). Handedness was assessed by means of a Dutch translation of the hand preference questionnaire of Annett (2004). Eight participants showed left-hand dominance. Participants had normal or corrected-to-normal vision and were paid for their participation. Participants were naïve concerning the experimental objectives and setup and were not given feedback on their performance. Prior to the experiment, participants gave their informed consent in writing. The study has been approved by the institutional research ethics committee and has been performed in accordance with the ethical standards as laid down in the 1964 Declaration of Helsinki.

### Apparatus

**Fig. 1:**
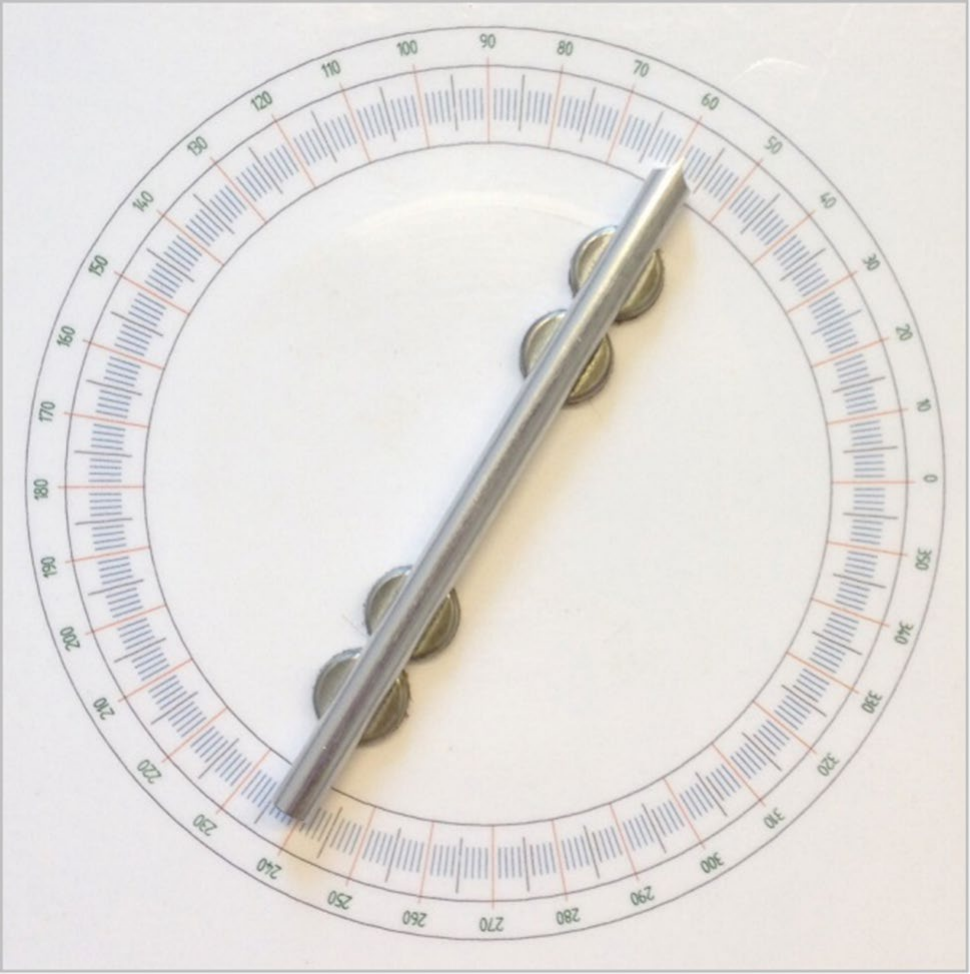
The protractor and the reference bar with the attached magnets

Two iron plates of 30 × 30 cm, which were covered with a plastic layer on which a protractor with a radius of 10 cm was printed, were used for the haptic parallel task. Each plate had an aluminum bar of 20 cm and a diameter of 1.1 cm (see Fig. 1). The bars had a small pin attached in the middle that fitted in a hole in the center of the protractor, making it possible to rotate the bars 360°. Small magnets were attached to the bottom side of the bars to increase their resistance to involuntary movement. To prevent accidental rotation of the reference bar, two extra magnets were attached under the reference bar (see also Fig. 1). Each bar had one side that ended in an arrow shape, making it possible to accurately read the orientation of both bars with a precision of about 0.5°.

**Fig. 2:**
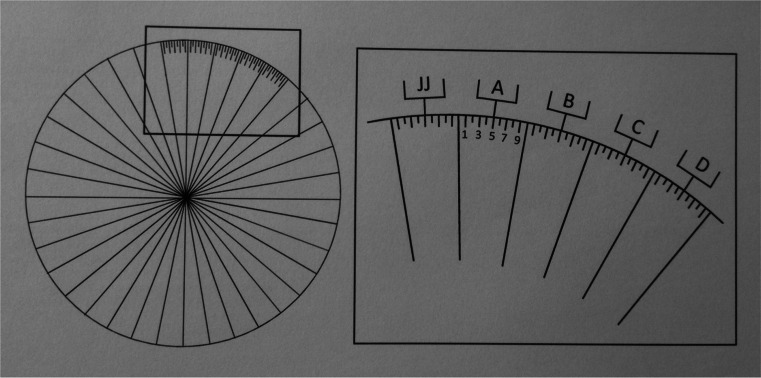
The test protractor containing *letters* and *numbers* that was used to “match” the orientations in the haptic visual parallelity task

To avoid that the plates would move or shift during the experiment, anti-slip mats were placed under each plate. In the haptic visual task, no test bar was used, but the protractor was replaced with a protractor in which the degrees were substituted by letters and numbers. This was done to avoid that participants would assume that certain orientations were used or would repeat previously reported orientations. This protractor was divided into 36 sections of 10° by lines starting in the middle of the protractor. Each section was marked with a letter, using the 26 letters of the alphabet, starting with single letters and ending with doubling of the letters, from A to JJ. The 36 sections were subdivided into 10 sections using only line marks on the circle of the protractor. Small mark lines were used for the even numbers and larger mark lines for the odd numbers (see Fig. 2). The odd numbers were added on the protractor. All 360° were represented by a letter and number, in such a way that, e.g., 90° was represented as JJ10 and 16° as H6.

### Experimental tasks

Participants performed two tasks, a haptic and a haptic visual task. In these tasks, the orientation of the reference bar was always explored with the non-dominant hand. Participants performed the haptic parallelity task (HP) while being blindfolded and had to rotate the test bar with their dominant hand in such a way that it felt parallel with respect to the reference bar. In the haptic visual task (HV), participants had full vision and were able to see everything except the plate with the reference bar and their exploring non-dominant hand, which were obstructed from view by a black box. In this task, participants had to feel the orientation of the reference bar and had to state which orientation on the test protractor was parallel to the felt orientation by verbally naming this orientation using the corresponding letter–number combination. In both tasks, the distance between the pivots of the bars, or center of the protractor, was set at 120 cm symmetrically around the midsagittal plane. Six different orientations were used 0°, 30°, 60°, 90°, 120° and 150°, with 0° being parallel to the horizontal edge of the table and increasing angular values signifying a rotation in counterclockwise direction. Each orientation was repeated three times. The order of orientations and repetitions was randomized within each task and for each participant in such a way that the same orientation was never repeated in consecutive trials.

### Procedure

Before the experiment started, the experimenter ensured that the participants had a clear understanding of parallelity by asking them to make two pens parallel to each other using different orientations. The haptic visual task was always presented last so that participants had not seen the setup during the haptic task.

In the haptic task, the experimenter placed the non-dominant hand of the participant above the reference bar and the dominant hand above the test bar. The task was performed bimanually, meaning that the bars were touched simultaneously. Participants were free regarding the positioning of their hands and the time needed to perform the task but were instructed not to touch (the sides of) the table or plates or to move their hands between the bars.

In the haptic visual task, the box that was placed over the plate with the reference bar was open at the side directed to the experimenter. That way the experimenter could easily orient the reference bar in one of the predetermined orientations outside the view of the participant. At the side of the participant, the box had an opening, covered by a cloth, through which the participant’s hand could be placed to feel the reference bar without seeing the plate with the bar and their forearm and hand. In this task, participants were instructed to place their dominant hand in front of them on the table and to keep it in a fixed position, to avoid that they would parallel both hands and use the orientation of their dominant hand to match it with the orientations on the test protractor. After each trial, the experimenter noted the orientation of the test bar or the letter–number combination given by the participant. The latter was later converted to the corresponding orientation.

## Results

The dependent variable was the smallest deviation between the orientation of the test bar and reference bar. Deviations that were clockwise to the orientation of the reference bar were noted as positive values, while negative values were assigned when the deviations were counterclockwise. This was reversed for the left-handed participants. In all analyses, signed deviations were used. The deviations were analyzed using a repeated measures ANOVA. First an overall analysis was done including repetition. Mean deviations were 32.2°, 31.3° and 31.6° for repetition 1–3. Because there was no significant main effect of repetition (*F*(2,116) = 0.64, *p* = .53) or any significant interactions with repetition, deviations were averaged over the three repetitions. The reported results are based on the repeated measures ANOVA with 3 factors, namely task (2: HP and HV) and orientation (6: 0°, 30°, 60°, 90°, 120° and 150°) as within-subject factors and gender (2) as between-subject factor. Gender effects were also separately studied per task. To test the effect of obliqueness, an additional analysis was performed in which the factor orientation was replaced by the factor obliqueness (average deviation of the cardinal orientations (0° and 90°) and of the oblique orientations (30°, 60°, 120° and 150°). In case of significant main effects, pairwise Bonferroni-corrected comparisons were computed. Only significant interaction effects are reported.

### Effect of task and orientation/obliqueness

The main effect of task was highly significant (*F*(1,58) = 141.60, *p* < .001). Mean deviations were 43.2° and 20.2° for the haptic parallel task (HP) and the haptic visual task (HV), respectively.

**Fig. 3:**
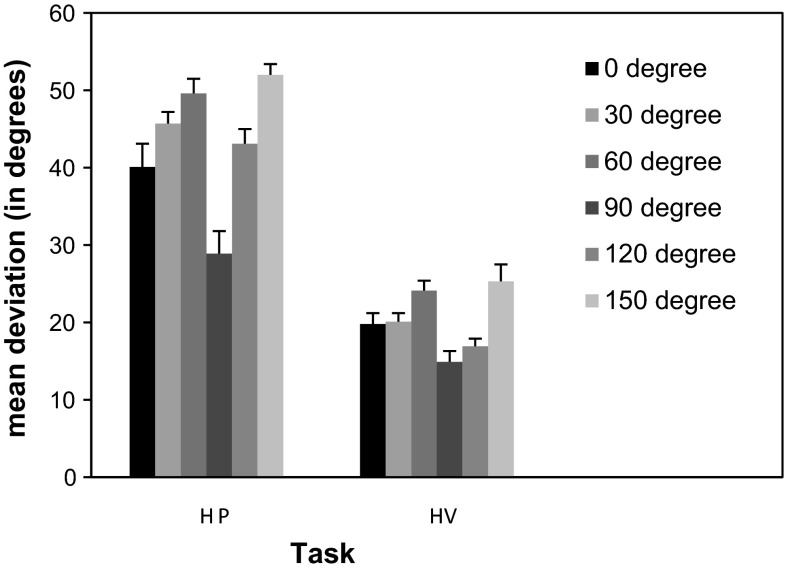
Mean deviations and standard errors for the different orientations in the tasks. *HP* haptic parallelity task, *HV* haptic visual parallelity task

As expected, the effect of orientation was also significant (*F*(5,290) = 25.10, *p* < .001). Deviations averaged over tasks were 29.9°, 32.9°, 36.8°, 21.9°, 30.0° and 38.6°, respectively, for the 0°, 30°, 60°, 90°, 120° and 150° orientations. Pairwise comparisons showed that the deviations for the vertical orientation of 90° were significantly smaller than the deviations for the other orientations (*p* < .001), while the deviations of the horizontal orientation of 0° were significantly smaller than the deviations for the 60° (*p* < .05) and the 150° (*p* < .001) orientation. A significant difference was also found between the deviations for the 60° and 150° orientations and between the 30° and 150° orientations (both *p* < .01). Additionally, the difference between the deviations of the 120° and 150° orientations was significant (*p* < .001). There was a significant interaction between task and orientation (*F*(5,290) = 5.77, *p* < .001). The latter was due to the fact that the deviations for the different orientations varied more in the haptic parallelity task than in the visual haptic task (see Fig. 3). However, separate analyses per task showed that the effect of orientation was significant in both tasks (*F*(5,290) = 22.04, *p* < .001 for task HP and *F*(5,290) = 8.32, *p* < .001 for task HV).

The additional analysis with obliqueness as factor revealed a significant effect for this factor (*F*(1,58) = 97.20, *p* < .001). The mean deviation for the cardinal orientations of 0° and 90° was 25.9°, while the mean deviation for the four oblique orientations was 34.6°. The interaction of task and obliqueness was also significant (*F*(1,58) = 12.12, *p* < .01). As stated above, this was due to the fact that differences in deviations between cardinal and oblique orientations were more pronounced in the haptic parallelity task (34.5° vs 47.6°) than in the haptic visual task (17.3° vs 21.6°). Separate analyses per task showed a significant effect of obliqueness in both tasks (*F*(1,58) = 31.99, *p* < .001 for task HP and *F*(1,58) = 8.81, *p* < .005 for task HV).

### Effect of gender

**Fig. 4:**
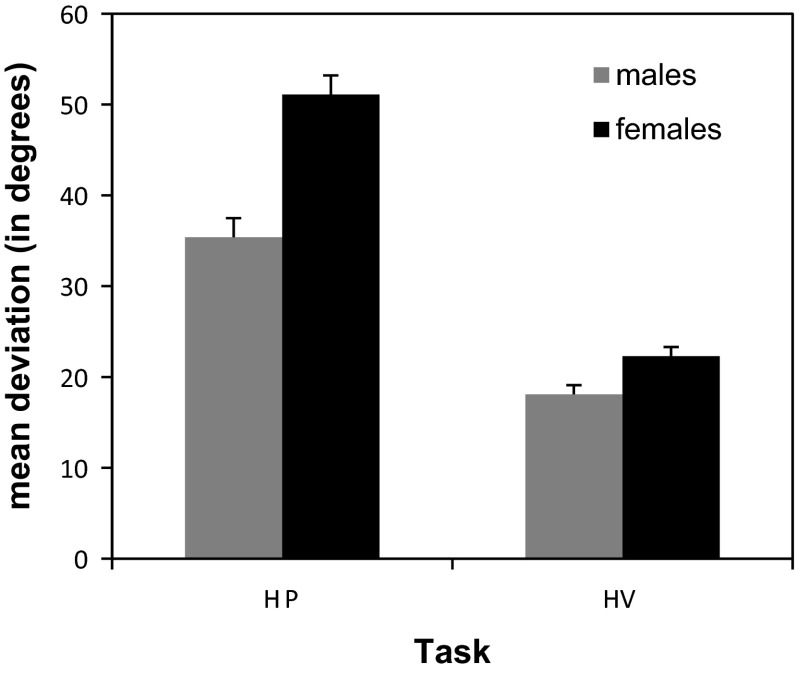
Mean deviations and standard errors for males and females in the tasks. *HP* haptic parallelity task, *HV* haptic visual parallelity task

The mean deviation over tasks was 26.7° for males and 36.7° for females, resulting in a significant main effect of gender (*F*(1,58) = 17.94, *p* < .001). Gender differences were not the same in both tasks, as suggested by the significant interaction of task and gender (*F*(1,58) = 8.81, *p* < .01). As can be seen in Fig. 4, gender differences were mainly found in the haptic parallelity task, in which males had a mean deviation of 35.4° and females of 51.1°. A separate analysis testing the effect of gender in the HP task revealed a significant main effect of gender (*F*(1,58) = 26.97, *p* < .001). No significant difference was found between the performance of males and females in the HV task, where males had a mean deviation of 18.1° and females of 22.3° (*F*(1,58) = 1.88, *p* = .18).

## Discussion

The aim of this study was to investigate whether eliminating or reducing the motor response when matching the orientation of a haptically perceived bar would abolish the gender difference observed in haptic parallelity matching. To this end, female and male participants had to haptically perceive the orientation of the reference bar (which was out of their view) and had to verbally report which line on the test plate matched the orientation of the reference bar.

Before discussing the results related to gender in the haptic visual task, the results of replicated findings in the haptic parallel task will be described. We found large and systematic deviations in the parallel task in line with previous reported results (e.g., Kappers 1999, 2003, 2004; Kaas and Van Mier 2006; Kaas et al. 2007b; Van Mier 2013). We replicated findings with respect to orientation and obliqueness. A significant effect of orientation, with larger deviations for oblique orientations than for cardinal orientations, was found in the haptic parallel task, consistent with the oblique effect reported by others (Kaas and Van Mier 2006; Kappers 2003; Kappers and Viergever 2006; Van Mier 2013; Volcic et al. 2007).

A significant effect of task was found. The largest deviations were observed in the haptic parallel task, while deviations in the haptic visual task showed a decrease in more than 50 % compared to the haptic parallel task. It seems that providing visual information, in the current experiment being able to see the protractor on the test plate, decreased deviations, as has been shown in other studies where vision was provided (Newport et al. 2002; Van Mier 2013; Volcic et al. 2008; Zuidhoek et al. 2004b). Being able to use visual information from the sides of the table and the plate with the protractor, and maybe also from the walls and doors, induced a shift to the use of a more allocentric reference frame. Additionally, because no physical test bar was available in the haptic visual task, participants had to mentally create a visual image of an oriented test bar. The use of visual imagery most likely also strengthened the influence of an allocentric reference frame (Postma et al. 2008; Zuidhoek et al. 2003, 2007). Deviations have also been observed in pure visual parallelity tasks, but were much smaller than in haptic tasks (Cuijpers et al. 2003; Kappers and Schakel 2011). In our haptic visual task, both modalities were mixed. Visual deviations, however, cannot be the result of a hand-centered egocentric bias; therefore, several factors might have influenced the results in our task. Knowing that haptic and visual space are both distorted with respect to physical space although differently (Cuijpers et al. 2003), our visual haptic task involved both spaces with their respective distortions. Cuijpers et al. (2003) described that matched settings in visual parallelity tasks also deviated from veridicality, as did the settings in haptic parallelity tasks, although deviations were much smaller in the visual task. The deviations depended linearly either on the separation angle between the bars (visual task) or the horizontal distance between the bars (haptic task). The authors stated that since the intrinsic geometries of visual and haptic space are Euclidean, both spaces can be matched. This might explain the comparable effects regarding the deviations for the different orientations in our haptic and haptic visual task.

When looking at the gender effect in the current study, we found as hypothesized that females had only significantly larger deviations than males in the haptic parallelity task, replicating previous reported results (Hermens et al. 2006; Kaas and Van Mier 2006; Kappers 2003, 2007; Van Mier 2013; Volcic et al. 2008; Zuidhoek et al. 2007). When no movement was required to parallel the orientation as in the haptic visual parallelity task, gender differences were eliminated. We rather use the term reducing hand movements of the dominant hand in the haptic visual task instead of eliminating hand movements, because we cannot completely rule out that participants did not move their dominant hand at all or used that hand as a reference. Sometimes a twitch or small movement could be seen in the participant’s dominant hand or the upper arm would be moved a little bit. However, these movements were in all cases very small and did not affect elimination of the gender difference. The findings regarding similar performance for both genders in our haptic visual parallelity task are in line with results reported by other parallelity studies in which no hand movement was required (Kappers and Schakel 2011), when reference or test bar were in the same location horizontally (Kappers and Liefers 2012), or when the egocentric bias of the hands was minimized (Van Mier 2013). Our results corroborate the idea that women use allocentric processing to the same extent as men (see also Van Gerven et al. 2012; Van Mier 2014) and profit from the use of an allocentric reference frame in parallel setting. However, when an orientation has to be paralleled involving both hands and when there is a horizontal distance between the hands, women seem to be less efficient overcoming the bias of the egocentric hand- and body-centered reference frame than men, resulting in larger deviations from parallelity in the former.

Elimination of the gender difference was not due to the informative vision that was provided in the haptic visual task in the current study. When Van Mier (2013) included informative vision in her study, by providing participants full view of the test bar and their matching hand, deviations were smaller than in the haptic parallel task for both genders, but females still had significantly larger deviations than males. It was suggested that performance of the women was biased by the use of the matching hand. Only when the bias of the hands was minimized by having participants draw the orientation (Van Mier 2013) or when matching of the orientation was done verbally without movement of the (test) hand, like in the haptic visual condition in the current study, the gender effect was abolished. So it is not vision per se that resulted in similar performance in both genders, but rather the reduction in the bias of the hand-centered egocentric reference frame by minimizing or reducing movements of the matching hand.

In conclusion, the results showed that haptic perception is susceptible to illusions; what participants felt as parallel was far from what was physically parallel, even when they could see the matching orientation in the haptic visual task. This illusion was significantly greater in women than in men, only in the haptic parallel task. As hypothesized, when movements of the matching hand were eliminated or reduced, this illusion was similar in both genders. Providing visual information in the haptic visual task while reducing the biasing influence of the hand-centered egocentric reference frame had a beneficial effect on haptic processing that was similar in both genders.

## References

[CR1] Annett M (2004). Hand preference observed in large healthy samples: classification, norms and interpretations of increased non-right-handedness by the right shift theory. Brit J Psychol.

[CR2] Cuijpers RH, Kappers AML, Koenderink JJ (2003). The metrics of visual and haptic space based on parallelity judgements. J Math Psych.

[CR3] Fernández-Díaz M, Travieso D (2011). Performance in haptic geometrical matching tasks depends on movement and position of the arm. Acta Psychol.

[CR4] Flanders M, Soechting JF (1995). Frames of reference for hand orientation. J Cogn Neurosci.

[CR5] Henriques DY, Flanders M, Soechting JF (2004). Haptic synthesis of shapes and sequences. J Neurophysiol.

[CR6] Hermens F, Kappers AML, Gielen SCAM (2006). The structure of frontoparallel haptic space is task dependent. Percept Psychophys.

[CR7] Kaas AL, Van Mier HI (2006). Haptic spatial matching in near peripersonal space. Exp Brain Res.

[CR8] Kaas AL, Van Mier HI, Goebel R (2007). The neural correlates of human working memory for haptically explored object orientations. Cereb Cortex.

[CR9] Kaas AL, Van Mier HI, Lataster J, Fingal M, Sack AT (2007). The effect of visuo-haptic congruency on haptic spatial matching. Exp Brain Res.

[CR10] Kappers AML (1999). Large systematic deviations in the haptic perception of parallelity. Perception.

[CR11] Kappers AML (2002). Haptic perception of parallelity in the midsagittal plane. Acta Psychol.

[CR12] Kappers AML (2003). Large systematic deviations in a bimanual parallelity task: further analysis of contributing factors. Acta Psychol.

[CR13] Kappers AML (2004). The contributions of egocentric and allocentric reference frames in haptic spatial tasks. Acta Psychol.

[CR14] Kappers AML (2007). Haptic space processing—allocentric and egocentric reference frames. Can J Exp Psychol.

[CR15] Kappers AML, Koenderink JJ (1999). Haptic perception of spatial relations. Perception.

[CR16] Kappers AML, Liefers BJ (2012) What feels parallel strongly depends on hand orientation. In: Isokosi P, Springare J (eds) Haptics: perception, devices, mobility, and communication, vol 7282 of Lecture notes on computer science. Springer, Berlin, pp 239–246

[CR17] Kappers AML, Schakel WB (2011). Comparison of the haptic and visual deviations in a parallelity task. Exp Brain Res.

[CR18] Kappers AML, Viergever RF (2006). Hand orientation is insufficiently compensated for in haptic spatial perception. Exp Brain Res.

[CR19] Klatzky RL, Freksa C, Habel C, Wender KF (1998). Allocentric and egocentric spatial representations: Definitions, distinctions, and interconnections. Spatial cognition: an interdisciplinary approach to representing and processing spatial knowledge.

[CR20] Newport R, Rabb B, Jackson SR (2002). Noninformative vision improves haptic spatial perception. Curr Biol.

[CR21] Postma A, Zuidhoek S, Noordzij ML, Kappers AML (2008). Haptic orientation perception benefits from visual experience: evidence from early blind, late blind and sighted people. Percept Psychophys.

[CR22] Van Gerven DJH, Schneider AN, Wuitchik DM, Skelton RW (2012). Direct measurement of spontaneous strategy selection in a virtual Morris water maze shows females choose an allocentric strategy at least as often as males do. Behav Neurosci.

[CR23] Van Mier HI (2013). Effects of visual information regarding allocentric processing in haptic parallelity matching. Acta Psychol.

[CR24] Van Mier HI (2014). Haptic perception of parallelity. Psychol Behav Sci.

[CR25] Volcic R, Kappers AML (2008). Allocentric and egocentric reference frames in the processing of three-dimensional haptic space. Exp Brain Res.

[CR26] Volcic R, Kappers AML, Koenderink JJ (2007). Haptic parallelity perception on the frontoparallel plane: the involvement of reference frames. Percept Psychophys.

[CR27] Volcic R, Van Rheede JJ, Postma A, Kappers AML (2008). Differential effects of non-informative vision and visual interference on haptic spatial processing. Exp Brain Res.

[CR28] Volcic R, Wijntjes MWA, Kappers AML (2009). Haptic mental rotation revisited: multiple reference frame dependence. Acta Psychol.

[CR29] Zuidhoek S, Kappers AML, Van der Lubbe RHJ, Postma A (2003). Delay improves performance on a haptic spatial matching task. Exp Brain Res.

[CR30] Zuidhoek S, Kappers AML, Noordzij ML, Van der Lubbe RH, Postma A, Ballesteros S, Heller MA (2004). Frames of reference in a haptic parallelity task: temporal dynamics and the possible role of vision. Touch, blindness and neuroscience.

[CR31] Zuidhoek S, Visser A, Bredero ME, Postma A (2004). Multisensory integration mechanisms in haptic space perception. Exp Brain Res.

[CR32] Zuidhoek S, Kappers AML, Postma A (2005). Effects of hand orientation and delay on the verbal judgment of haptically perceived orientation. Perception.

[CR33] Zuidhoek S, Kappers AML, Postma A (2007). Haptic orientation perception: sex differences and lateralization of functions. Neuropsychologia.

